# Roles of Toll-Like Receptor 3 in Human Tumors

**DOI:** 10.3389/fimmu.2021.667454

**Published:** 2021-04-27

**Authors:** Xin Zheng, Song Li, Hui Yang

**Affiliations:** Department of Neurosurgery, Xinqiao Hospital, Army Medical University, Chongqing, China

**Keywords:** Toll-like receptor 3 - TLR-3, tumors, opposing role, dsRNA, pathogen-associated molecular pattern

## Abstract

Toll-like receptor 3 (TLR3) is an important member of the TLR family, which is an important group of pathogen-associated molecular patterns. TLR3 can recognize double-stranded RNA and induce activation of NF-κB and the production of type I interferons. In addition to its immune-associated role, TLR3 has also been detected in some tumors. However TLR3 can play protumor or antitumor roles in different tumors or cell lines. Here, we review the basic signaling associated with TLR3 and the pro- or antitumor roles of TLR3 in different types of tumors and discuss the possible reasons for the opposing roles of TLR3 in tumors.

## Introduction

The link between cancer and inflammation has been firmly established by epidemiological investigations and *in vivo* and *in vitro* studies (1). Nearly 25% of cancers are attributable to chronic inflammation triggered by infection or physicochemical agents (2). Pattern recognition receptors (PRRs) can detect nonself or altered molecular patterns that act as important upstream regulators to control inflammation. Toll-like receptors (TLRs) are the best characterized PRRs that detect pathogen-associated molecular patterns (PAMPs) (3). Activation of TLRs triggers multiple proinflammatory and other signaling cascades to eliminate pathogens. However, if pathogens cannot be eliminated, they may elicit chronic inflammation though TLR signaling (4). In addition to PAMPs, TLRs recognize damage-associated molecular patterns (DAMPs), which are proteins or nucleic acids released during necrosis. Cell death results in the release of numerous DAMPs, such as heat-shock proteins (HSPs), fibrinogen, and endogenous double-stranded (ds) and single-stranded RNA. Once these molecules and substances are released from necrotic cells, they can activate TLRs expressed on other cells, which subsequently upregulate NF-κB expression and release cytokines and chemokines (5).

The TLR family is composed of 10 members (TLR1-TLR10) in humans (6). TLRs are characterized by extracellular leucine-rich repeat (LRR) domains that recognize pathogens, a transmembrane domain for cellular localization and an intracellular Toll/IL-1 receptor (TIR) domain for the transduction of downstream signals (7). Different TLR members have specific subcellular localizations and agonists. TLR 1, -2, -4, -5, -6, and -10 are cell surface proteins. TLR3 and TLR7-9 are located inside the cell in endocytic compartments and the endoplasmic reticulum. Surface TLR members respond to various bacterial lipoproteins, glycolipids, lipopolysaccharides and flagella. The intracellular proteins recognize oligonucleotides from self and microbial origins [for more details on TLR ligands, see review (8) and (9)].

Like other TLRs, TLR3 is predominantly expressed in innate immune cells such as macrophages, dendritic cells (DCs) and natural killer (NK) cells and are thus involved in the activation of innate immunity, which results in the production of proinflammatory cytokines, chemokines, and adhesion molecules (10). Intriguingly, increasing evidence has demonstrated that TLR3 is also expressed in tumor cells. The activation of TLR3 in both tumor cells and the tumor microenvironment, such as in typical innate immune cells, might play an opposing role in tumor progression.

In this review, we focus on the “double-edged sword” roles of TLR3 in tumor initiation and progression. We summarize the mechanism and clinical trials associated with the role of TLR3 in tumor behavior. In addition, we discuss the possible reasons for the dual roles of TLR3 in tumors.

## Basic Structure and Classic Signaling Pathways of TLR3

TLR3 is normally located in endosomes, where its luminal ectodomain (ECD) encounters its ligands, most important of which is dsRNA. The TLR3-ECD is composed of 23 LRRs and resembles a long solenoid bent into the shape of a horseshoe (11). The TLR3-ECD horseshoe is flat and highly glycosylated. The flatness of the TLR3 horseshoe facilitates ligand binding and signaling (12). The binding site of TLR3 is specific for dsRNA but does not distinguish between base sequences of dsRNA ligands. TLR3-ECDs interact with dsRNA at two sites on the glycan-free lateral surface. These sites interact only with the ribose-phosphate backbone of the dsRNA, which leads to nonspecific base sequence binding of the TLR3 ligand (13). The minimum length required for dsRNA binding to TLR3 is 40-45 bp, and the optimal pH for dsRNA binding to TLR3-ECD is below 6.5 (13, 14). The affinity of TLR3 for dsRNA is high (~10 nM) under optimal conditions but decreases as the pH increases above 6 and the dsRNA length decreases below 45 bp (13).

Once dsRNA binds to the TLR3-ECD, the adaptor protein TIR domain-containing adapter inducing interferon-b (TRIF or TICAM1) is recruited *via* a TIR-TIR domain interaction. Then, downstream signaling molecules access their binding sites upon colocalization of TRIF and TLR3 (15, 16). TRIF recruits downstream signaling molecules such as NAK-associated protein 1 (NAP1), tumor necrosis factor receptor-associated factor 3 (TRAF3), IRF-3-activating kinases, TANK-binding kinase 1 (TBK1) and IκB kinase (IKK-cc), which bind to the N-terminal region of TRIF. This results in activation, phosphorylation and dimerization of IRF3 and IRF7, which induce the secretion of type I IFNs (17).

TLR3 can also activate NF-κB in a TRIF-dependent manner *via* receptor interacting protein 1 (RIP1) (18). Upon activation of TLR3, RIP1 is recruited to TRIF, where it is phosphorylated and polyubiquitinated by Peli1, an E3 ubiquitin ligase, which leads to the formation of a complex involving TRAF6, the TAK1-binding protein TAB2, the ubiquitin-activated MAP3K, TAK1 and the dsRNA-dependent kinase PKR (19, 20). This complex then translocates to the cytoplasm. Then, TAK1 is phosphorylated and activated, which results in formation of the IKK complex, which is composed of IKKa, IKKb, NEMO and NF-κB (19, 21, 22). Activation of NF-κB induces the expression of genes that maintain cell proliferation, promote invasion, and activate inflammatory cytokines. TAK1 can also activate MAP kinase signaling pathways by phosphorylating MKK6 (19). TRIF can recruit RIP3 and RIP1. The RIP1-RIP3 complex can play synergistic roles in cell survival. RIP1 can recruit FADD and induce caspase 8-mediated apoptosis. At the same time, RIP3 blocks RIP1-induced NF-κB-mediated cell survival (23). RIP1 and RIP3 can both participate in the generation of reactive oxygen species (ROS), which induce cell apoptosis and necrosis (24, 25).

## Roles of TLR3 in Human Cancers

TLR3 is expressed in certain types of cancers (**Table 1**) that are often related to viral infection. TLR3 is closely related to clinical characteristics, prognosis and the probability of metastasis. However, the conclusions from *in vitro* or mouse model studies vary greatly.

**Table 1:** TLR3 expressions in human cancers.

Cancer Type	Tissue or Cell lines	TLR3 roles on cancer	Ref
Cervical cancer	human cancer tissues	early carcinogenesis	(26)
	U14 cell line	protumor	(27)
	Hela cell line	antitumor	(28)
	mouse model	immune adjuvants	(29)
Hepatocellular carcinoma	human cancer tissues	expressed more in large tumors	(30)
	human cancer tissues	antitumor	(31)
	HepG2.2.15 cell line	antitumor	(32)
	MHCC97H cell line	antitumor	(33)
	SMMC-7721 cell line	antitumor	(33, 34)
	HUVEC cell line	antitumor	(33)
	mouse model	antitumor	(35)
Multiple myeloma	KMM1 cell line	protumor	(36)
	NCI-H929 cell line	antitumor	(36)
	RPMI8226 cell line	antitumor	(36)
	B16 implantation model	antitumor	(37)
Melanoma	B16F10 mouse model	antitumor	(38)
	C57BL/6 mouse model	antitumor	(39)
Breast cancer	human cancer tissues	related to recurrence and metastasis	(40)
	Cama-1 cell line	antitumor	(41)
	BT-483 cell line	antitumor	(41)
	SW527 cell line	antitumor	(41)
	MCF-7 cell line	surface stimulation of TLR3: protumor	(42)
	MCF-7 cell line	cytoplasmic stimulation of TLR3: antitumor	(42)
	MDA-MB-231 cell line	surface stimulation of TLR3: protumor	(42)
	MDA-MB-231 cell line	cytoplasmic stimulation of TLR3: antitumor	(42)
	MDA-MB-231 cell line	antimetastatic effect	(43)
	MDA-MB-231 cell line	antitumor	(44)
Prostate cancer	human cancer tissues	related to recurrence	(45, 46)
	LNCaP cell line	antitumor	(47, 48)
	PC3 cell line	antitumor	(49)
	DU145 cell line	antitumor	(49)
Head and neck cancers	human cancer tissues	related to poorly differentiated tumors	(50)
	PCI1 cell line	protumor	(50)
	BHY cell line	protumor	(50)
	Detroit-562 cell line	protumor	(51)
	OC2 cell line	protumor	(52, 53)
	HEp2 cell line	antitumor	(54)
	SCC25 cell line	antitumor	(54)
	HSC2、3、4 cell line	antitumor	(54)
	SAS	antitumor	(54)
	HSQ89	antitumor	(54)
	HO-1-u-1	antitumor	(54)
	HB	antitumor	(55)
	CAL27	antitumor	(55)
	WSU-HN6	antitumor	(55)
	YD-10B	antitumor	(56)
	YD-8	antitumor	(56)

### Cervical Cancer

High-risk human papillomavirus (HPV) types that are related to the development of epithelial abnormalities of the cervix were identified as a necessary cause of cervical cancer (57). TLR3 is related to the clearance of HPV in healthy populations (58). HPV has also been demonstrated to modulate TLR expression and signaling, thus leading to persistent viral infection and carcinogenesis (59). DeCarlo et al. compared the expression of TLR3 among normal, premalignant and malignant cervical tissues and found that TLR3 expression was increased in dysplastic but not carcinomatous epithelium and that TLR3 mRNA levels were not changed in the carcinomatous stroma. This finding indicates that TLR3 is involved in early cervical carcinogenesis (26). In an *in vitro* study using the cervical cancer cell line U14, treatment with TLR3-siRNA significantly decreased cell growth, migration and invasion. This finding indicates the role of TLR3 in the development of cervical cancer (27). Polyinosinic-polycytidylic acid [i.e. poly(I:C)] is a synthetic analog of double-stranded RNA (dsRNA). Upon binding to its receptors, poly(I:C) is able to selectively activate numerous signaling pathways depending on the conditions. Poly(I:C) can be recognized by endosomal TLR3, thus usually considered as the agonist of TLR3. However, in the HeLa cell line, the TLR3 ligand poly (I:C) can induce apoptosis though IFN-β production (28). Poly (I:C) can be an important adjuvant that enhances tumor killing. In a cervical cancer mouse model, poly (I:C) treatment after administration of the HPV E6/E7 oncogene-specific vaccine IVAG increased vaccine-specific IFN-γ-secreting CD8 T cells by 5-fold (29). Pretreatment with E7(44-62) and poly (I:C) can increase TNF-α and IFN-γ secretion in NK92 cells and stimulate human DCs to secrete CD11c and CD86, which ultimately increases cytotoxicity against HeLa cells (60).

### Hepatocellular Carcinoma

The leading causes of hepatocellular carcinoma (HCC) are chronic infections with hepatitis B (HBV) and hepatitis C viruses (HCV) worldwide (61). In HCC tissues, the positive detection rate of TLR3 varies from 52.7% to 91%, and TLR3 is expressed in both the membrane and cytoplasm. Surface stimulation of TLR3 did not affect cell viability even when NF-κB was activated. However, cytoplasmic stimulation can induce apoptosis by downregulating the expression of antiapoptotic proteins (62, 63). Eiro et al. found that TLR3 levels were significantly higher in large tumors than in small tumors (30). However, TLR3 expression both in tumor cells and intratumoral cells correlates with prolonged survival (64–66). TLR3 was expressed at lower levels in HCC tissues than in adjacent tissues. Survivin, Bcl-2 and MMP2 expression were negatively correlated with TLR3 expression, but caspase 3, -8, and -9 were positively correlated with TLR3 signaling proteins in human HCC tissues (31).

In an *in vitro* study, the TLR3 tended to have antitumor roles in different HCC cell lines. HBV secretion by the HepG2.2.15 cell line can be inhibited by dsRNA-mediated activation of TLR3 (67). In addition, the TLR3 agonist BM-06 can inhibit proliferation and invasion and simultaneously promote apoptosis of HepG2.2.15 cells by activating NF-κB (32). The downstream factors Caspase 8, Bcl-2, and PCNA were all affected (68). Guo et al. found that the TLR3 agonists BM-06 and poly (I:C) can trigger apoptosis in MHCC97H, SMMC-7721 and HUVEC cell lines and inhibit cell migration (33). As TLR3 can both activate NF-κB and IRF3, Khvalevsky et al. observed short-term IRF3 activation and a low level of IFN-β in the Huh7 HCC cell line after TLR3 activation. In the HepG2 cell line, no induction of proinflammatory factors was observed, but apoptosis was induced *via* TLR3 activation (35). In the SMMC-7721 cell line, the combination of poly (I:C) and ATO can inhibit cell growth by increasing ROS generation and inducing mitochondrial dysfunction, in addition to upregulating caspase 3/8/9 and Bax and downregulating Bcl-2, survivin and Bid (34). In an HCC mouse model, poly (I:C)-induced TLR3 signaling can lead to a reduction in tumor enlargement and decrease tumor growth and cell proliferation (69, 70). In addition, in a spontaneous liver tumor mouse model, poly (I:C) treatment can increase remodeling of the tumor microenvironment, including intratumoral chemokine expression, NK cell activation and infiltration and proliferation of tumor-infiltrating T cells (64).

### Multiple Myeloma

Many studies have investigated the roles of TLR3 in multiple myeloma (MM). There are many established myeloma cell lines. Jego et al. found that TLR3 was expressed in 5 of 16 investigated cell lines (NCI-H929, SBN, XG1, RPMI8226, and KMS11) (71). In addition, Abdi et al. found that the Fravel, L363, UM6, UM9, OPM1, OPM2 and U266 cell lines also expressed TLR3 (72). Among these TLR3-expressing myeloma cell lines, the effects of poly (I:C) stimulation are not consistent. A study by Chiron et al. showed that even though XG1 and OPM2 cells express TLR3, they did not respond to poly (I:C). Poly (I:C)-mediated activation of NF-κB was observed in NCI-H929, RPMI8226 and KMM1 cells, which express TLR3. After stimulation, the proliferation of KMM1 cells was increased, but the proliferation of NCI-H929 and RPMI8226 cells was decreased. Apoptosis of the latter two cell lines was due to activation of the IFN-α-p38-MAPK pathway and ERK1/2 pathway, which are involved in cell death (36). Apoptosis in myeloma can also be induced through the TLR3/TRIF/caspase-8 pathway (73), TLR3/IFN-β pathway (74) and TLR3/IFN-α/Bcl-2 pathway and caspase-3, caspase-9, X-IAP, cFLIP, and A20 (75). Immune cells can efficiently affect myeloma cells *via* stimulation with TLR3 agonists. In a B16 tumor implantation model, poly (I:C) can activate mDC-derived antitumor NK cells, which led to the retardation of tumor growth (37).

### Melanoma

TLR3 is highly expressed on human melanoma cells, and TLR3 activation can induce activation of downstream NF-κB and cell migration (76). In a poorly immunogenic B16-OVA melanoma tumor model, poly (I:C) was used an adjuvant that combined with anti-CD40 and efficiently induced tumor rejection (77). These antitumoral effects must occur in combination with the induced immunochemotherapeutic regimen of vaccination against tumor antigens (78). In the B16F10 mouse model, poly (I:C) liposomes can inhibit melanoma growth in a dose-dependent manner, accompanied by increasing numbers of TRP-2-tetramer(+)CD8(+) cells in lymph nodes, maturation of DCs and increasing numbers of TRP1-specific IFN-γ-producing cells in lymph nodes and the spleen (38). Poly (I:C) can also inhibit the metastasis of myeloma cells. In a B16 melanoma cell C57BL/6 mouse model, intraperitoneal injection of poly (I:C) can inhibit lung and liver metastasis of B16 cells in a manner dependent on NK cells and IFN-γ, along with increasing the number of IFN-producing killer DCs in the spleen, lung and liver (39).

### Breast Cancer

IFN-γ is considered a key factor associated with the immune dysfunction that is common in breast cancer. Amarante et al. found that among patients with high expression of IFN-γ, TLR3 mRNA expression was significantly higher in breast cancer tissues than in healthy tissues, while TLR3 protein expression did not differ between healthy and breast cancer tissues (79). In contrast, Gonzalez et al. found that the expression level of TLR3 was related to recurrence and an increased probability of metastasis (40). In a randomized clinical trial, adjuvant treatment with poly(A:U) dsRNA was associated with a significant decrease in the risk of metastatic relapse in TLR3(+) breast cancer patients but not in TLR3(-) patients (80).

In an *in vitro* study, activation of TLR3 by dsRNA can trigger apoptosis in human breast cancer cells, which involves Toll/IL-1R domain-containing adaptors that induce IFN-β and type I IFN autocrine signaling, as well as activation of extrinsic caspases (41). Interestingly, a study by Bondhopadhyay et al. used MCF-7 and MDA-MB-231 breast cancer cell lines and found that surface stimulation of TLR3 can increase cellular survivability, growth, E-cadherin expression and thus metastasis. However, cytoplasmic stimulation led to a decrease in cell survival (42). Another TLR3 agonist, poly (A:U), did not have cytotoxic or apoptotic effects on the MDA-MB-231 cell line but decreased the expression of MMP2 at high concentrations, which indicated an antimetastatic effect (43). Galli et al. found that after treatment with poly (I:C), microRNA (miR)29b, miR29c, miR148b, and miR152 were increased in MDA-MB-231 cells. These microRNAs led to demethylation and re-expression of the oncosuppressor RARβ, thus inducing apoptosis (44). TLR3 stimulation can also result in the induction of breast cancer stem cells (CSCs). TLR3 stimulation can trigger coactivation of NF-κB and β-catenin, which promote breast cancer cells to acquire a CSC phenotype *in vivo* and *in vitro (* 81).

### Prostate Cancer

Prostate cancer tissues exhibited higher expression levels of TLR3 than healthy tissues, which was also related to a higher probability of biochemical recurrence (45). Schulz et al. also found that the expression of TLR3 was associated with prostate cancer recurrence (46). *In vitro*, the prostate cancer cell lines LNCaP and DU-145 can respond to TLR3 agonists through NF-κB activation, but the more aggressive PC3 cell line cannot. After activation of TLR3, LNCaP and DU-145 cells upregulated inflammatory molecules and cytokines that attracted immune effectors (47). In the LNCaP cell line, poly (I:C) induced intrinsic and extrinsic apoptotic pathways through interferon regulatory factor-3 signaling (48), and the PI3K/Akt pathway was inactivated, accompanied by the participation of the autophagy-, proliferation- and apoptosis-related proteins cyclin D1, c-Myc, p53 and NOXA (82). Mitogen-activated protein kinases were activated by poly (I:C) in both LNCap and PC3 cells. In addition, the interferon-independent pathway p38 and c-jun N-terminal kinase/protein kinase C α were also activated in the LNCaP cell line (83). Surprisingly, in PC3 cells, activation of TLR3 can increase the expression of the specific I.3 isoform of HIF-1α, which results in reduced apoptosis and VEGF secretion; that is, TLR3 plays an antitumor role. In the LNCaP cell line, activation of TLR3 cannot induce nuclear accumulation of HIF-2 α (49). A study by Palchetti et al. focused on the role of the TLR3/Scr/STAT1 axis in apoptosis in PC3 and DU145 cell lines (84).

### Head and Neck Cancers

Head and neck cancers also express TLR3. Approximately 73.2% of oral squamous cell carcinoma (OSCC) tissues express TLR3, and TLR3 expression is correlated with poorly differentiated tumors. TLR3 can activate NF-κB/c-Myc and increase the proliferation of head and neck squamous cell carcinoma (HNSCC) cells. TLR3 was considered to contribute to the malignant phenotype that leads to invasion (50). In the HNSCC cell line Detroit-562, activation of TLR3/NF-κB can upregulate the expression of ICAM-1, IL-6, IL-8, and IL-1β and promote cell migration but decrease cell viability (51). TLR3 activation can induce metabolic reprogramming and lead to increased aerobic glycolysis and cell migration though changes in the expression of pyruvate kinase, CD44 variants and MMP9 in Detroit 562 cell line (52). Stimulation of the TLR3-expressing OC2 cell line (a head and neck cancer cell line) with poly (I:C) can induce the phosphorylation of IRF3 and IκB, ultimately increasing the secretion of IL-6 and CCL5. CCL5 can promote the migration of OC2 cells (53). TLR3 may contribute to tumor development and lead to cisplatin resistance in OC2 cells (54). However, in 8 other head and neck cancer cell lines (HEp2, SCC25, HSC2, HSC3, HSC4, SAS, HSQ89 and HO-1-u-1), poly (I:C)-stimulated TLR3 induced apoptosis in these cell lines though downregulating survivin expression (55). In addition, in the OSCC cell lines HB, CAL27 and WSU-HN6, poly (I:C) induced apoptosis *via* TLR3 though the production of IFN-β and activation of caspase 3 and 9 (56). In the OSCC cell lines YD-10B and YD-8, apoptosis was induced *via* a mitochondria-dependent pathway (85).

### Other Tumors

In colorectal cancer, TLR3 is considered a marker of colon tissue metaplasia. The normal colonic mucosa expresses the highest levels of TLR3. As the malignancy stage increases from polyp to stage I to III adenocarcinoma, the expression of TLR3 decreases (86, 87). *In vitro*, poly (I:C) can induce necroptosis in the colon cancer cell line CT26 though the TLR3-TICAM-1-RIP3 axis to produce reactive oxygen (88). In mouse colon tumor models, the combination of poly (I:C) and vaccination with the CEA epitope can cause increase the number of mature DCs and induce elevated expression IL-12 and proliferation of lymphocytes (89). TLR3 is highly expressed in human gastric cancer tissues and is correlated with worse clinical characteristics such as poor overall survival, lymph node invasion and poor prognosis (90). However, in neuroblastomas, positive TLR3 expression is associated with favorable histology and prognosis (91). In normal renal tissues, TLR3 expression is limited. In clear cell renal cell carcinoma (CCRCC), TLR3 is highly expressed and correlated with lung metastasis (92). TLR3 is also highly expressed in nonsmall cell lung cancer and esophageal squamous cells compared with that of healthy tissues (93, 94). Activation of lung alveolar epithelial TLR3 by tumor exosomal RNAs can recruit neutrophils, which can promote the formation of the lung premetastatic niche (95). However, activation of TLR3 in the Lewis lung cancer cell line 3LL induces M1 polarization of tumor-associated macrophages and can inhibit tumor growth (96). The inhibitory role of TLR3 has been are verified in LL/2 and A549 NSCLC cells (97). The expression of TLR3 can be considered a biomarker for lymph node metastasis and increased depth of invasion of esophageal squamous cell carcinoma (94). In pituitary adenomas (PAs), TLR3 is expressed in every PA sample and correlated with the invasion and proliferation of PAs. In an *in vitro* study, activation of TLR3 in the rat pituitary adenoma GH3 cell line induced proliferation, invasion and secretion of inflammatory cytokines (98).

## Clinical Applications of TLR3-Based Immune Adjuvants

Tumor vaccines are used to treat and protect against tumors. The innate immune response is important in supporting the development of adaptive immunity and thus can be used to accelerate and enhance induction of vaccine-specific responses (99). Stimulation with poly (I:C) can result in strong type I and III interferon and Th1 cytokine responses. Type I interferon production is considered to be critical for efficacy of poly (I:C) as a Th1-inducing adjuvant (100), which can impact APC maturation, antigen processing and the responding antigen-specific B and T cells (101, 102). As high doses of poly (I:C) have been shown to cause shock, renal failure and coagulopathies in Phase I-II clinical trials of cancer patients (103), two safer derivatives of poly (I:C) were produced, poly-ICLC (Hiltonol^®^) and poly-IC_12_U (Ampligen^®^).

Poly-IC_12_U has been shown to induce functional, mature DCs in cancer patients (104). In Phase I and Phase II clinical trials for leukemia and neuroblastoma cancer patients, poly-ICLC elicited strong interferon responses and was partially successful in inducing tumor regression (105, 106). In addition, poly-ICLC also enhanced antibody titers and CD4 and CD8 T cell responses in an ovarian cancer clinical trial (107). Poly-IC_12_U was well tolerated in long-term Phase I-II clinical trials involving cancer patients (108). High-dose poly-ICLC therapy was associated with hypotension, fever and anemia. However, once the dose and frequency of delivery were optimized, the treatment was be well tolerated in patients (106, 109, 110). A defined TLR3-specific adjuvant, which is a chimeric molecule consisting of phosphorothioate ODN-guided dsRNA, was synthesized that can’t activate the RIG-1/MDA5 pathway. It elicited far less cytokine production than poly(I:C) *in vitro* and *in vivo* (111).

## TLR3 Diversity in Different Tumors

The expression of TLR3 in cancer cells seems to be quite common, but the roles and molecular mechanism of TLR3 in cancers are quite complicated. The downstream factors NF-κB and IRF3 are two important transcription factors associated with TLR3 signaling (6). Activation of NF-κB can induce inflammatory cytokine and MMP secretion, which may promote cancer progression (112). However, activation of IRF3 can induce the production of type I interferon, which can result in cancer cell apoptosis (17). These two different pathways may be the basis of the different outcomes of TLR3 activation in cancer cells. Overall, TLR3 tends to play an antitumor role, but its protumor role should be mentioned in clinical applications.

TLR3 is normally located in endosomes. Cell surface TLR3 expression has been detected in fibroblasts, vascular endothelial cells and epithelial cells (113). In some immune cells, cell surface expression of TLR3 has also been detected. TLR3 is not expressed on the cell surface of human monocyte-derived DCs (114), but an established mAb against mouse TLR3 was used to show that TLR3 was highly expressed on the cell surface of splenic CD8^+^ DCs and marginal zone B cells. TLR3 is also expressed on the surface of macrophages, and even though its expression is weak, it can enhance TLR3 responses to dsRNA (115). Activation of cell surface TLR3 leads to a proinflammatory response, whereas TLR3 activation in endosomal compartments by dsRNA is characterized by type I interferon production and plays an anti-inflammatory role (113). In different types of tumors, the expression of TLR3 varies greatly. There is not enough information on cell surface expression of TLR3 in different tumors. This may be the reason why in different cancers, the effects of TLR3 can be varied. In addition, in the same kind of cancer, the role of TLR3 also differs in diverse cell lines, as we previously mentioned.

## Conclusion

In further studies on TLR3, the role of cell surface TLR3 should not be ignored. Cell surface TLR3 expression should be measured and localized by immunohistochemistry or other methods. The downstream signaling of TLR3 should be comprehensively considered, both the NF-κB pathway and the IRF3/7 pathway. In related TLR3 clinical trials, the dual role of TLR3 should be considered, and activation of TLR3 may increase the progression of some kinds of tumors or in some individuals. Further mechanistic investigations on the dual roles of TLR3 in tumor biology are needed.

## Author Contributions

All authors listed have made a substantial, direct, and intellectual contribution to the work and approved it for publication.

## Funding

This work was supported by National Natural Science Foundation of China (No. 81801369).

## Conflict of Interest

The authors declare that the research was conducted in the absence of any commercial or financial relationships that could be construed as a potential conflict of interest.
